# Extra Spindle Pole Bodies-Like 1 Serves as a Prognostic Biomarker and Promotes Lung Adenocarcinoma Metastasis

**DOI:** 10.3389/fonc.2022.930647

**Published:** 2022-06-22

**Authors:** Zhi Nie, Tong Pu, Zhaojie Han, Chenyang Wang, Chenglong Pan, Ping Li, Xiaoling Ma, Yanfei Yao, Youmei Zhao, Chunyan Wang, Xiulin Jiang, Jianyang Ding

**Affiliations:** ^1^Department of Neurology, First Affiliated Hospital of Kunming Medical University, Kunming, China; ^2^Department of Neurology, Yunnan Province Clinical Research Center for Neurological Diseases, Kunming, China; ^3^Key Laboratory of Animal Models and Human Disease Mechanisms of Chinese Academy of Sciences and Yunnan Province, Kunming Institute of Zoology, Kunming, China; ^4^The College of Acupuncture and Tuina and Rehabilitation, Hunan University of Chinese Medicine, Changsha, China; ^5^Department of Thoracic Surgery, Southwest Hospital, Army Medical University, Chongqing, China; ^6^Department of Pathology, First Affiliated Hospital of Kunming Medical University, Kunming, China; ^7^Department of Anatomy and Histology/Embryology, Faculty of Basic Medical Sciences, Kunming Medical University, Kunming, China; ^8^Department of Cardiothoracic Surgery, The People’s Hospital of Lishui, Lishui, China

**Keywords:** extra spindle pole bodies-like 1, lung adenocarcinoma, prognosis biomarker, DNA methylation, immune infiltration

## Abstract

Extra spindle pole bodies-like 1 (ESPL1), a cysteine endopeptidase, plays a vital role in chromosome inheritance. However, the association of ESPL1 with prognosis and immune infiltration in lung adenocarcinoma (LUAD) has not yet been explored. Here, we analyzed the expression level, prognostic values, diagnostic value, and immune infiltration level in LUAD using various databases. Immunohistochemistry (IHC) and quantitative real-time PCR (qRT-PCR) assays were used to detect the expression of ESPL1 in LUAD tissues and cell lines. In this study, we found that ESPL1 was upregulated in LUAD and a higher expression of ESPL1 was correlated with unfavorable prognosis in LUAD. Meanwhile, Cox hazard regression analysis results suggested that ESPL1 may be an independent prognostic factor for LUAD. Moreover, we demonstrated that ESPL1 expression was significantly correlated with immune infiltration of Th2 and dendritic cells in LUAD. We also confirmed that DNA copy number amplification and DNA hypo-methylation were positively correlated with ESPL1 expression in LUAD. Additionally, DNA copy number amplification was significantly associated with adverse clinical outcomes in LUAD. Kyoto Encyclopedia of Genes and Genomes (KEGG) and gene set enrichment analysis (GSEA) confirmed that ESPL1 was mainly involved in the DNA replication and glycolysis signaling pathway. Finally, we revealed that ESPL1 was highly expressed in LUAD tissues and cell lines. Knockdown of ESPL1 significantly inhibited cell migration and the invasion abilities of LUAD. Our study comprehensively confirmed that ESPL1 expression may serve as a novel prognostic biomarker for both the clinical outcome and immune cell infiltration in LUAD.

## Introduction

Lung cancer is the leading cause of cancer-related death worldwide, according to Cancer Statistics 2022 (1). The incidence rate of lung cancer ranks second, while the death rate of lung cancer ranks first (2). Lung cancer includes small cell lung carcinoma (SCLC) and non-small cell lung carcinoma (NSCLC). NSCLC includes lung adenocarcinoma (ADC), lung squamous cell carcinoma (SCC), and large-cell lung carcinoma. The NSCLC cancer accounts for approximately 85% of all cases (3). With the development of medical technology, a variety of treatment methods have emerged, while the 5-year survival rate and prognosis of LUAD patient remain disappointing (4). Immune checkpoint inhibitors targeting programmed cell death protein 1 (PD1) or programmed death ligand 1 (PD-L1) have already substantially improved the outcomes of patients with many types of cancer, but only 20%–40% of patients benefit from these therapies (5). Therefore, it will be helpful for improving the effect of immunotherapy to find the indicators of immune infiltration and explore its possible mechanism.

ESPL1, a cysteine endopeptidase, plays an important role in modulating the chromosome inheritance (6). It has been confirmed that ESPL1 was overexpressed in glioma and associated with the pathological features and poor prognostics in glioma patients (7). Jiang et al. found that ESPL1 was higher in chronic HBV infection patients and maybe as a biomarker for screening HBV-related hepatocellular carcinoma (HCC) and monitoring recurrence (8). Studies reported that ESPL1 was elevated in endometrial cancer, and its higher expression correlated with late stage and higher tumor grade (9). However, the upstream regulatory mechanisms, the correlation between ESPL1 and clinical outcomes, and immune cell infiltration in LUAD patients remain unclear.

In the present study, we determine the expression pattern, prognostic value, and diagnostic value of ESPL1 by using The Cancer Genome Atlas (TCGA) and the Gene Expression Omnibus (GEO) datasets. Functional gene/protein association network and functional enrichment were analyzed by GeneMANINA and STRING databases. The TIMER and TISIDB databases were used to examine the potential relationship of ESPL1 expression with immune cell infiltration in LUAD, and Spearman’s correlation was used to evaluate samples from the TIMER and GEPIA databases. IHC and qRT-PCR assay were used to detect the expression of ESPL1 in LUAD tissues and cell lines. Transwell and wound healing assays were used to determine the potential function of ESPL1 on LUAD cell migration and invasion abilities.

## Materials and Methods

### ESPL1 Expression Level and Prognosis Analysis

ESPL1 expression data and patient clinical data were downloaded from the TCGA data portal (website: https://www.cbioportal.org/; dataset: TCGA, LUAD) (10). In this study, we used it to analyze the association between ESPL1 expression and clinical features. Meanwhile, we also explored the diagnostic value and prognostic value of ESPL1 in LUAD. We used the Human Protein Atlas (HPA: https://www.proteinatlas.org/) database to determine the protein level of ESPL1 in lung cancer and normal tissues (11).

### PrognoScan Database Analysis

The PrognoScan database (http://www.abren.net/PrognoScan/) (12), including various GEO datasets in this study, was used to determine the overall survival of ESPL1 in different lung cancer-related GEO datasets. The threshold was Cox *p*-value < 0.05.

### LinkedOmics Database

LinkedOmics (http://www.linkedomics.org/login.php) is a publicly available portal that includes the multi-omics data of TCGA pan-cancer. In this study, we used to determine the genes that were correlated with ESPL1 in LUAD.

### Gene–Gene and Protein–Protein Interaction Network of ESPL1

We constructed the gene–gene and protein–protein interaction network of ESPL1 by using the STRING database (https://string-db.org/) and GeneMANIA (http://www.genemania.org) (13, 14).

### CancerSEA Database

CancerSEA (http://biocc.hrbmu.edu.cn/CancerSEA/) is the first dedicated database that aims to comprehensively explore distinct functional states of cancer cells at the single-cell level. CancerSEA portrays a cancer single-cell functional state atlas, involving 14 functional states (including stemness, invasion, metastasis, proliferation, EMT, angiogenesis, apoptosis, cell cycle, differentiation, DNA damage, DNA repair, hypoxia, inflammation, and quiescence) of 41,900 single cancer cells from 25 cancer types (15). In this study, we used it to determine the distinct functional states of ESPL1 at the single-cell level in LUAD.

### DNA Copy Number Variation and DNA Methylation Analysis

Gene Set Cancer Analysis (GSCA) is an integrated platform, including the genomic (expression, SNV, CNV, and methylation) and clinical information (16). In this study, we used it to examine the relationship between ESPL1 expression and CNV and DNA methylation in LUAD. Meanwhile, we used it to determine the prognostic value of ESPL1 CNV in LUAD. UALCAN (http://ualcan.path.uab.edu) is a database that includes the gene expression and survival analyses based on the TCGA dataset (17). In this study, we used it to analyze the DNA methylation level of ESPL1 in LUAD.

### Immune Cell Infiltration Analysis

TIMER (https://cistrome.shinyapps.io/timer/) is a database that includes the gene expression and immune cell infiltrations in human cancers (18); in this study, we used to determine the correlation between ESPL1 CNV and immune cell infiltrations in LUAD. We also used the GSVA R package to quantify the LUAD immune infiltration of 24 tumor-infiltrating immune cells in LUAD samples *via* ssGSEA based on the 509 gene signatures of 24 tumor-infiltrating lymphocytes (TILs) (19).

### Cell Culture

The BEAS-2B cell line was purchased from the Chinese Academy of Sciences Cell Bank (CASCB, China), and cultured in BEGM media (Lonza, CC-3170). Lung cancer cell lines, including H358, H1650, A549, and H1299, were purchased from CASCB with STR documents, and were cultured in RPMI-1640 medium (Corning) supplemented with 10% fetal bovine serum (FBS) and 1% penicillin/streptomycin.

### Constructs, Transfection, Infection, and qPCR Assay

Independent shRNAs targeting ESPL1 were synthesized and cloned into the lentiviral plasmid pLKO.1. The lentiviruses were generated according to the manufacturer’s protocol. Total RNA was extracted according to the manufacturer’s protocol, and then reverse transcribed using an RT reagent kit. The primers used in this study are as follows: β-actin-F: AAGTGTGACGTGGACATCCGC and β-actin-R: CCGGACTCGTCATACTCCTGCT; and ESPL1-F: CCGCCTTGAAGGAGTTCCTG and ESPL1-R: GGGGTAGACACTAAGTAGCCAT.

### Cell Migration and Invasion Assay

Cell migration and invasion assay was performed as previously documented (20). For transwell assay, 2.5×10^4^ cells in 100 μl of serum-free medium were plated in a 24-well plate chamber insert, with medium containing 10% FBS at the bottom of the insert. Cells were incubated for 24 h, and then fixed with 4% paraformaldehyde for 20 min. After washing, cells were stained with 0.5% crystal violet blue. The positively stained cells were examined under the microscope.

### Immunohistochemical Staining

For immunohistochemical staining, the sections were deparaffinized in xylene and rehydrated through graded ethanol. Antigen retrieval was performed for 20 min at 95°C with sodium citrate buffer (pH 6.0). After quenching endogenous peroxidase activity with 3% H_2_O_2_ and blocking non-specific binding with 1% bovine serum albumin buffer, sections were incubated overnight at 4°C with the indicated primary antibodies. Following several washes, the sections were treated with HRP-conjugated secondary antibody for 40 min at room temperature, and stained with 3,3-diaminobenzidine tetrahydrochloride (DAB).

### Statistical Analyses

All statistical analyses were performed using GraphPad Prism 7.0, and ROC curves were used to detect ESPL1 cutoff values using pROC packages. *p*-values were calculated by either unpaired or paired two-tailed Student’s *t*-test, *p < 0.05, **p < 0.01, and ***p < 0.001.

## Results

### Expression Pattern and Prognostic Value of ESPL1 in Pan-Cancers

**Figure 1:**
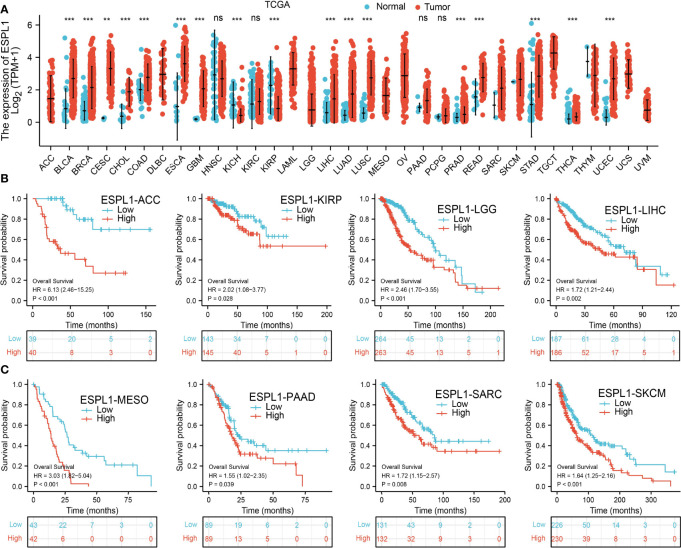
The expression level and prognosis of ESPL1 in pan-cancer. **(A)** Pan-cancer expression of ESPL1 between tumor tissues from the TCGA database. **(B, C)** Kaplan–Meier overall survival of ESPL1 in pan-cancers. NS: *p* > 0.05, **p < 0.01; ***p < 0.001.

We first determine ESPL1 expression in TCGA pan-cancer. Results suggested that a higher ESPL1 expression was observed in 13 tumors: BLCA, BRCA, CESC, CHOL, COAD, ESCA, GBM, LIHC, LUAD, LUSC, PRAD, READ, and UCEC (**Figure 1A**).

Furthermore, we explore the prognostic significance of ESPL1 in various human cancer types. Results show that ESPL1 is a risk factor for ACC, KIRP, LGG, LIHC, MEAO, PAAD, SARC, and SKCM (**Figures 1B, C**).

### ESPL1 Was Upregulated in Lung Adenocarcinoma

**Figure 2:**
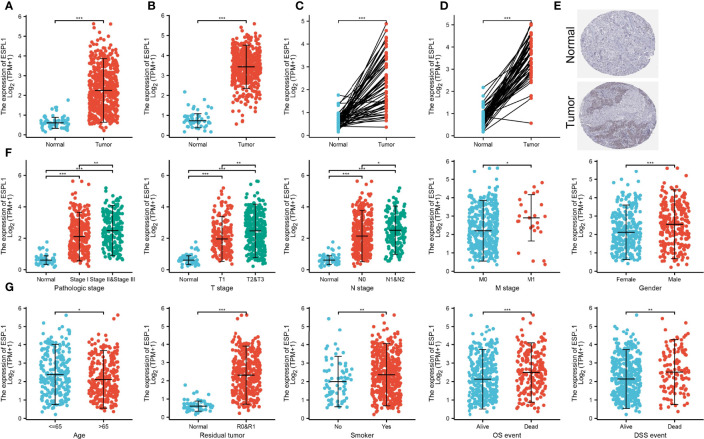
Correlation between ESPL1 mRNA expression and clinicopathological parameters. **(A–D)** ESPL1 was upregulated in LUAD and LUSC based on the TCGA database. **(E)** Protein level of ESPL1 in lung cancer tissue examined by the HPA database. **(F, G)** Correlation between ESPL1 expression and clinical stage, TNM stage, gender, age, residual tumor, smoker, OS event, and DSS event in patients with LUAD. *p < 0.05; **p < 0.01; ***p < 0.001.

We further found that ESPL1 was highly expressed in LUAD and LUSC than adjacent normal tissues based on the TCGA database (**Figures 2A, D**). Moreover, IHC results confirmed that ESPL1 was upregulated in lung cancer tissues compared with normal lung tissues (**Figure 2E**). Given the crucial role of ESPL1 in LUAD, we examined the potential correlation between ESPL1 expression and clinical features, including multiple clinicopathological characteristics and survival of LUAD patients. Results suggested that higher expression of ESPL1 was significantly correlated with higher clinical stage, TNM stage, gender, age, residual tumor, smoker, OS event, and DSS event in patients with LUAD (**Figures 2F, G** and **Table 1**).

**Table 1:** Correlation between ESPL1 expression and clinicopathological features in TCGA-LUAD.

Characteristic	Low expression of ESPL1	High expression of ESPL1	*p*
* n*	267	268	
T stage, *n* (%)			0.003
T1	107 (20.1%)	68 (12.8%)	
T2	128 (24.1%)	161 (30.3%)	
T3	24 (4.5%)	25 (4.7%)	
T4	7 (1.3%)	12 (2.3%)	
N stage, *n* (%)			0.002
N0	187 (36%)	161 (31%)	
N1	43 (8.3%)	52 (10%)	
N2	24 (4.6%)	50 (9.6%)	
N3	0 (0%)	2 (0.4%)	
M stage, *n* (%)			0.016
M0	184 (47.7%)	177 (45.9%)	
M1	6 (1.6%)	19 (4.9%)	
Pathologic stage, *n* (%)			<0.001
Stage I	168 (31.9%)	126 (23.9%)	
Stage II	56 (10.6%)	67 (12.7%)	
Stage III	30 (5.7%)	54 (10.2%)	
Stage IV	7 (1.3%)	19 (3.6%)	
Gender, *n* (%)			<0.001
Female	165 (30.8%)	121 (22.6%)	
Male	102 (19.1%)	147 (27.5%)	

### Knockdown of ESPL1 Inhibits Lung Cancer Cell Migration and Invasion

**Figure 3:**
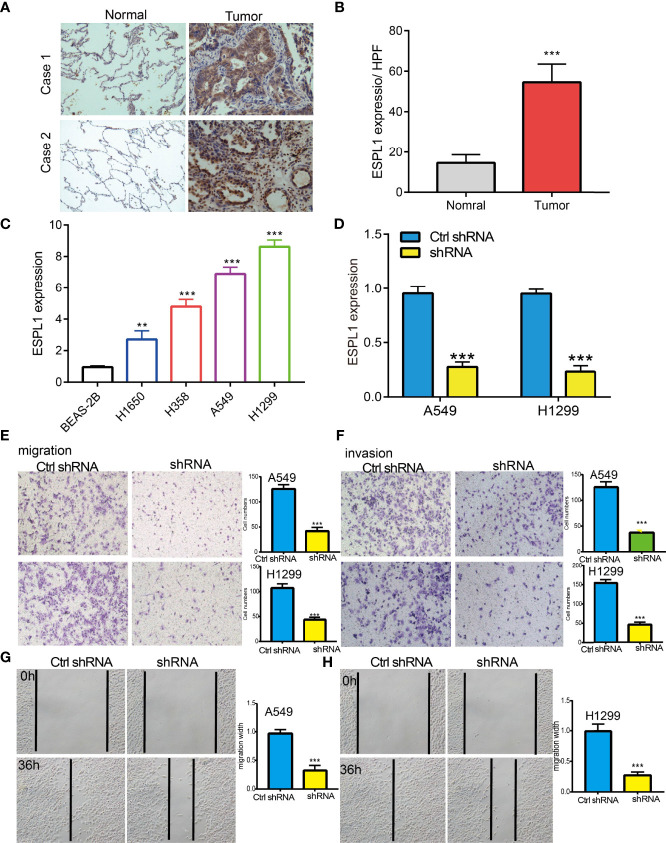
ESPL1 knockdown inhibited cell migration and invasion of LUAD. **(A, B)** IHC assay used to determine the protein of ESPL1 in clinical lung cancer samples. **(C)** The relative expression of ESPL1 in LUAD cell lines including H1650, H358, A549, and H1299 examined by real-time RT-PCR and human bronchial epithelial (BEAS2B) cell line was used as control. **(D)** Establishment of ESPL1 knockdown in A549 and H1299 cells, verified by real-time RT-PCR. **(E–H)** Downregulation of ESPL1 inhibited A549 and H1299 cell migration and invasion by transwell and wound healing assays, Quantification data were also indicated for each assay. Scale bar = 50 μm. **p < 0.01; ***p < 0.001.

IHC assay confirmed that ESPL1 was upregulated in lung cancer tissues compared with normal lung tissues (**Figures 3A, B**). We also found that ESPL1 was increased in LUAD cell lines compared with the normal lung epithelial cell line (BEAS2B) (**Figure 3C**), which is consistent with the online database we discovered. Given that ESPL1 was upregulated in LUAD, we then inhibited the ESPL1 expression using shRNA, and knockdown efficiency of ESPL1 was verified by real-time RT-PCR assay (**Figure 3D**). Then, we evaluated the effects of ESPL1 on LUAD cell migration and invasion capacities by wound healing and transwell migration assays. We showed that downregulation of ESPL1 significantly decreased the migratory and invasion capabilities of LUAD cells (**Figures 3E–H**). Collectively, these results confirmed that ESPL1 was highly expressed in LUAD cells and significantly affected their migration and invasion.

### Analysis of the Diagnostic and Prognostic Value of ESPL1 in LUAD

**Figure 4:**
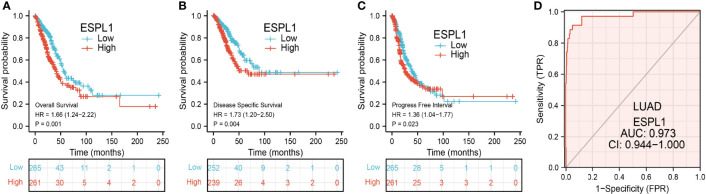
Prognostic and diagnostic values of ESPL1 in LUAD. **(A–C)** Correlation between ESPL1 expression and OS, DSS, and PFS in patients with LUAD examined by the TCGA datasets. **(D)** ROC curve analysis of the diagnostic values of ESPL1 in LUAD.

**Figure 5:**
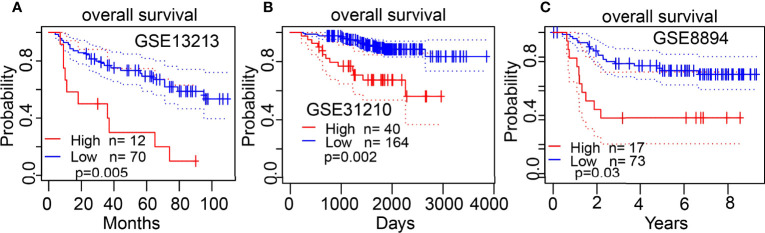
Validation of the overall survival of ESPL1 in LUAD. **(A–C)** Correlation between ESPL1 expression and OS in patients with lung cancer examined by GEO datasets.

The Kaplan–Meier curve method was conducted to examine the relationship between ESPL1 expression level and overall survival (OS), disease-specific survival (DSS), and progression-free survival (PFS) in patients with LUAD. We found that patients in the higher ESPL1 class had a shorter probability of OS, DSS, and PFS compared to the low ESPL1 group (**Figures 4A–C**). Then, receiver operating characteristic (ROC) curve analysis suggested that the ESPL1 may be used to differentiate LUAD patients from normal control (AUC = 0.973) (**Figure 4D**). Moreover, we also investigated the prognostic role of ESPL1 across several independent GEO clinical datasets. Results suggested that upregulated ESPL1 expression was associated with adverse clinical outcomes in patients with lung cancer (**Figures 5A–C**).

### Clinical Stratification

**Figure 6:**
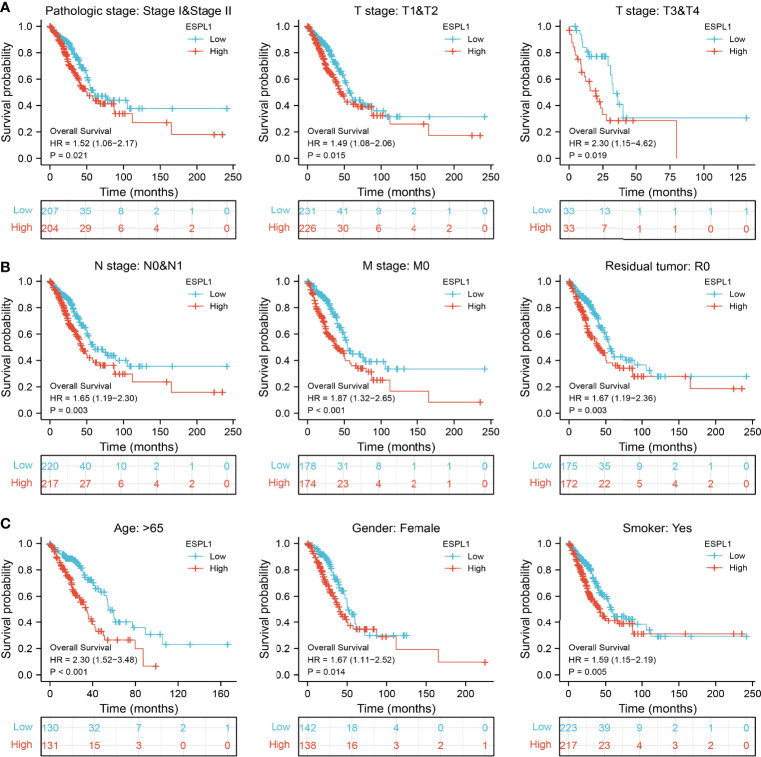
Validation of the overall survival of ESPL1 in diverse clinical subtypes. **(A–C)** Validation of the overall survival of ESPL1 in diverse clinical subtypes, including stage I–II, T1/T2, T3/T4, N0/N1, M0, R0, age>65, female, and smoker.

We also determine the overall survival of ESPL1 in different clinical subgroups, including stage I–II, T1/T2, T3/T4, N0/N1, M0, R0, age>65, female, and smoker. Results confirmed that lower ESPL1 expression had better survival outcomes than those with highly expressing ESPL1 in patients with LUAD (**Figures 6A–C**). Logistic regression analysis suggested that T stage (T2 and T3 and T4 vs. T1), N stage (N1 and N2 and N3 vs. N0), M stage (M1 vs. M0), pathologic stage (Stage III and Stage IV vs. Stage I and Stage II), and gender (Male vs. Female) were significantly correlated with ESPL1 expression in LUAD patients (**Table 2**). To examine the potential prognostic factors for OS and DSS of LUAD patients, univariate regression analysis and a multivariate model have shown significant prognostic significance of ESPL1 expression, pathological stage, and TNM stage for OS and DSS (**Tables 3**, **4**).

**Table 2:** Logistic regression analyzed the correlation between ESPL1 expression and clinicopathological characteristics in LUAD.

Characteristics	Total (*N*)	Odds Ratio (OR)	*p*-value
T stage (T2 and T3 and T4 vs. T1)	532	1.959 (1.358–2.842)	<0.001
N stage (N1 and N2 and N3 vs. N0)	519	1.803 (1.245–2.624)	0.002
M stage (M1 vs. M0)	386	3.292 (1.357–9.211)	0.013
Pathologic stage (Stage III and Stage IV vs. Stage I and Stage II)	527	2.290 (1.484–3.584)	<0.001
Gender (Male vs. Female)	535	1.965 (1.394–2.779)	<0.001
Age (>65 vs. <=65)	516	0.733 (0.518–1.036)	0.079

**Table 3:** Univariate regression and multivariate survival model of overall survival in patients with LUAD.

Characteristics	Total (*N*)	Univariate analysis		Multivariate analysis	
		Hazard ratio (95% CI)	*p*-value	Hazard ratio (95% CI)	*p*-value
Pathologic stage	518				
Stage I and Stage II	411				
Stage III and Stage IV	107	2.664 (1.960–3.621)	<0.001	1.927 (1.177–3.155)	0.009
T stage	504				
T1	175				
T2 and T3	329	1.658 (1.175–2.341)	0.004	1.643 (1.050–2.570)	0.030
N stage	508				
N0	343				
N1 and N2	165	2.645 (1.977–3.539)	<0.001	1.728 (1.136–2.627)	0.011
M stage	377				
M0	352				
M1	25	2.136 (1.248–3.653)	0.006	1.004 (0.460–2.191)	0.992
Gender	526				
Female	280				
Male	246	1.070 (0.803–1.426)	0.642		
ESPL1	526	1.233 (1.090–1.395)	<0.001	1.162 (1.125–1.354)	0.0453

**Table 4:** Univariate regression and multivariate survival model of disease-specific survival in patients with LUAD.

Characteristics	Total (*N*)	Univariate analysis		Multivariate analysis	
		Hazard ratio (95% CI)	*p*-value	Hazard ratio (95% CI)	*p*-value
Pathologic stage	483				
Stage I and Stage II	389				
Stage III and Stage IV	94	2.436 (1.645–3.605)	<0.001	1.319 (0.700–2.484)	0.392
T stage	473				
T1	168				
T2 and T3	305	1.815 (1.169–2.819)	0.008	1.652 (0.936–2.914)	0.083
N stage	473				
N0	327				
N1 and N2	146	2.755 (1.909–3.975)	<0.001	2.059 (1.230–3.446)	0.006
M stage	344				
M0	323				
M1	21	2.455 (1.269–4.749)	0.008	1.782 (0.734–4.327)	0.202
Gender	491				
Female	262				
Male	229	0.989 (0.687–1.424)	0.954		
ESPL1	491	1.285 (1.097–1.504)	0.002	1.219 (1.006–1.478)	0.044

### CNV and DNA Methylation Analysis

**Figure 7:**
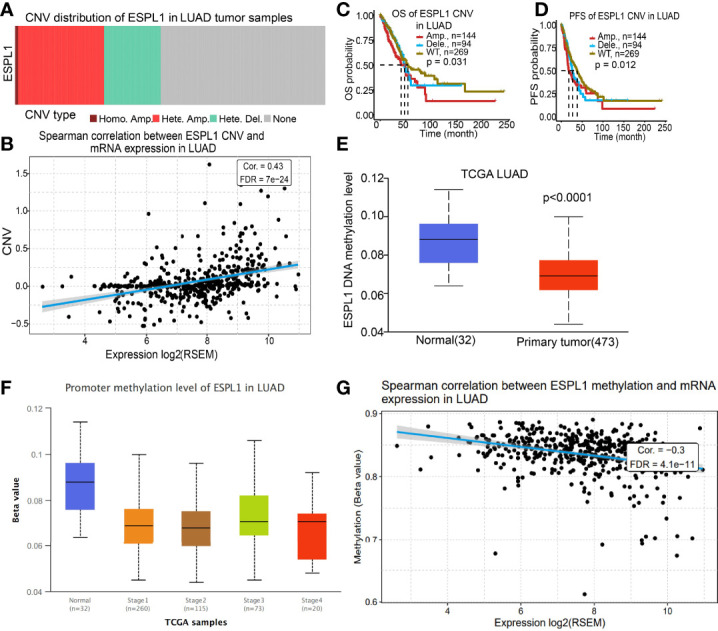
CNV and DNA methylation analysis. **(A, B)** Correlation between ESPL1 expression and CNV of ESPL1 in LUAD. **(C, D)** Correlation between prognosis and CNV of ESPL1 in LUAD examined by the TCGA database. **(E)** DNA methylation level of ESPL1 in LUAD. **(F)** Correlation between pathological stage and DNA methylation of ESPL1 in LUAD examined by the TCGA database. **(G)** Correlation between ESPL1 expression and DNA methylation in LUAD examined by the TCGA database.

Given that CNV and DNA methylation play crucial roles in modulating gene expression and are involved in cancer progression (21), we used the GSCA database to determine the correlation between CNV and DNA methylation and ESPL1 expression in LUAD. We found that CNV may be the reason for ESPL1 overexpression in LUAD (**Figure 7A**). We also confirmed a positive correlation between CNV and ESPL1 mRNA expression in LUAD (**Figure 7B**). Next, we assessed the prognostic value of ESPL1 CNV in terms of LUAD and overall survival. Results confirmed that the ESPL1 copy-number-altered group was associated with poorer OS and PFS in LUAD (**Figures 7C, D**). Then, we further investigated the potential association between DNA methylation and ESPL1 expression. We utilized the UALCAN database to explore the DNA methylation level of ESPL1 in human cancer. Results showed that in LUAD, ESPL1 DNA methylation level was significantly lower in tumor tissues than in normal tissues (**Figure 7E**). Meanwhile, we revealed that ESPL1 DNA methylation levels significantly decreased in accordance with the progression of LUAD stage I to stage II (**Figure 7F**). We also demonstrated a negative correlation between DNA methylation and ESPL1 expression in LUAD (**Figure 7G**).

### Identification of ESPL1-Interacting Genes and Proteins and Single-Cell Functional Analysis

**Figure 8:**
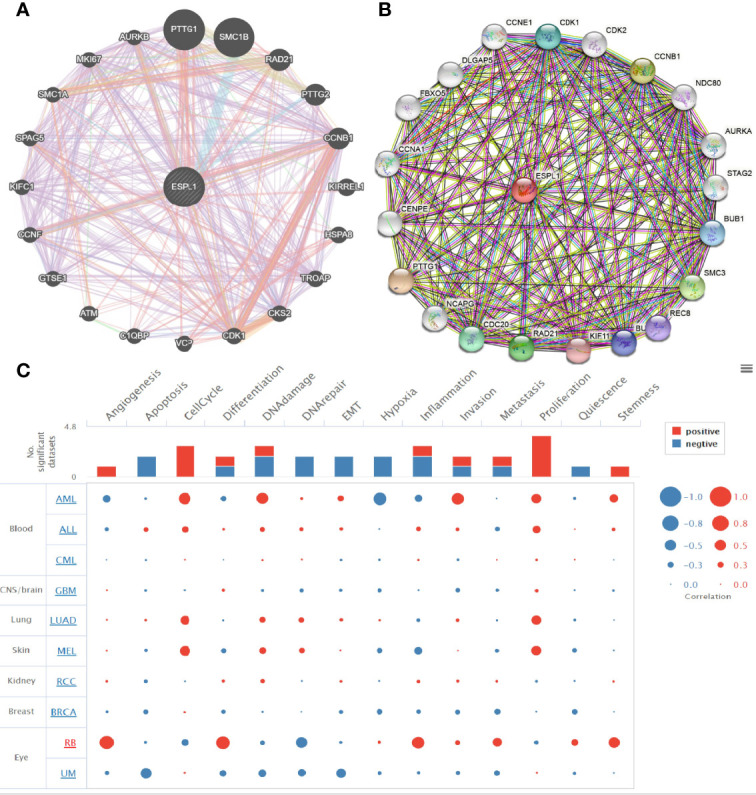
Gene–gene and protein–protein interaction network. **(A, B)** Gene–gene and protein–protein interaction network constructed by the genemania and STRING databases. **(C)** CancerSEA used to explore the potential role that ESPL1 might play in single LUAD cells.

We used the GeneMania database to construct the gene–gene interaction network of ESPL1. Results suggested that the 20 most frequently altered genes were closely correlated with ESPL1, including PTTG1, SMC1B, and CCNB1 (**Figure 8A**). A protein–protein interaction (PPI) network of ESPL1 was generated by using the STRING database. We found that 20 proteins were closely correlated with ESPL1, including CDK1, CDK2, CCNB1, NDC80, SMC3, and BUB1 (**Figure 8B**). Next, we used CancerSEA, a single-cell database to explore the potential role that ESPL1 might play in single LUAD cells. Results suggested that ESPL1 was found to be mainly involved in cell cycle, DNA damage, EMT, DNA repair, and cell proliferation (**Figure 8C**).

### KEGG and GSEA Enrichment Analysis

**Figure 9:**
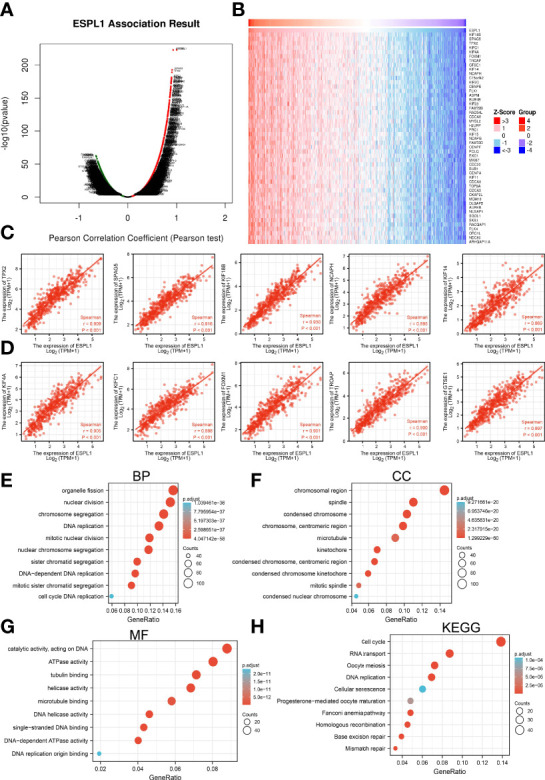
KEGG enrichment analysis of ESPL1. **(A–D)** Identified genes with positive co-expression with ESPL1 using the TCGA database. **(E–H)** GO and KEGG enrichment analysis of ESPL1 in LUAD.

We identified genes with positive co-expression with ESPL1 using the TCGA database and obtained the top 300 genes that are positively correlated with ESPL1 in LUAD (**Figures 9A–D**). To determine the potential molecular function by which ESPL1 modulates LUAD progression, we conducted Kyoto Encyclopedia of Genes and Genomes (KEGG) and Gene Ontology (GO) enrichment using the 300 genes that were positively related to ESPL1 in pan-cancers, respectively. As shown in **Figures 9E–H**, for the biological process, these genes were mainly enriched in chromosome segregation, DNA replication, and mitotic nuclear division (**Figure 9E**). For the cellular component, these genes were mainly enriched in chromosomal region, spindle, condensed chromosome, and microtubule (**Figure 9F**). For the molecular function, these genes were mainly enriched in ATPase activity, tubulin binding, helicase activity, and DNA helicase activity (**Figure 9G**). Moreover, KEGG pathway analysis suggested that ESPL1 was associated with signaling pathways related to the cell cycle, RNA transport, DNA replication, and cellular senescence (**Figure 9H**).

**Figure 10:**
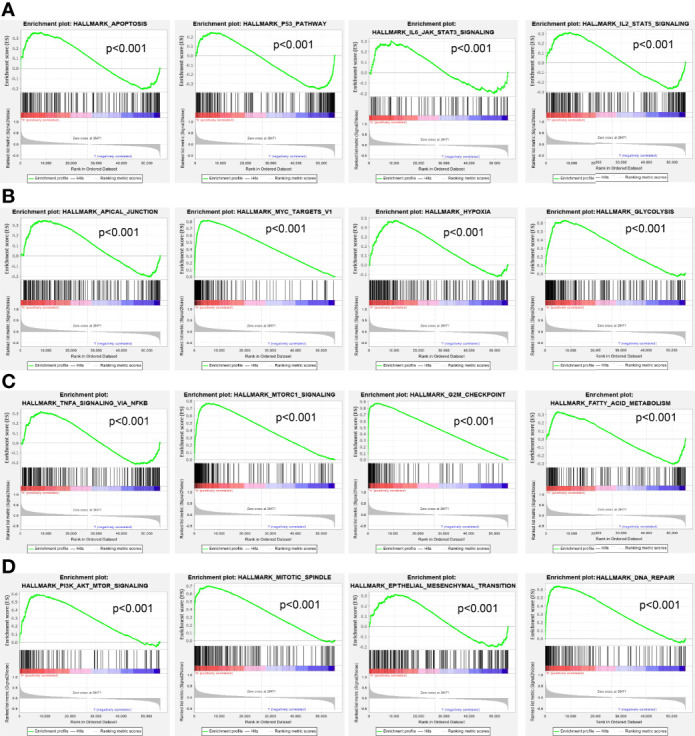
GSEA Identification of ESPL1-related signaling pathways. **(A–D)** GSEA identification of ESPL1-related signaling pathways in LUAD.

Moreover, GSEA was performed to determine the activated signaling pathways correlated with ESPL1 in LUAD. The signaling pathways activated in ESPL1 overexpressed phenotype, including apoptosis, p53 signaling pathway, IL6-JAK-STAT3 signaling pathway, IL3-STAT5 signaling pathway, MYC targets, hypoxia, glycolysis, TNFα signaling pathway, G2/M checkpoint, fatty acid metabolism, PI3K-AKT signaling pathway, EMT, and DNA repair (**Figures 10A–D**).

### Immune Analysis of ESPL1 in LUAD

**Figure 11:**
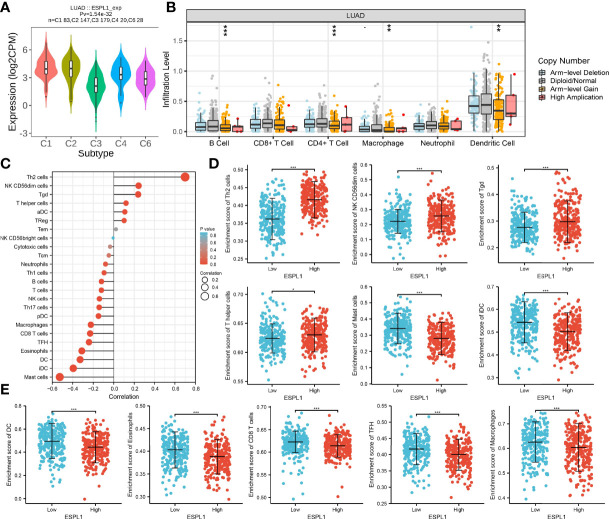
Correlation analysis of ESPL1 expression and infiltration levels of immune cells in LUAD. **(A)** ESPL1 expression in diverse immune subtypes. **(B)** Correlation analysis of ESPL1 CNV and infiltration levels of immune cells in LUAD. **(C–E)** Correlation analysis of ESPL1 expression and infiltration levels of immune cells in LUAD. *p < 0.05; **p < 0.01; ***p < 0.001.

Next, the association between ESPL1 expression and tumor immune infiltration was analyzed. Results indicated that ESPL1 expression was significantly upregulated in the C2 subtype of LUAD (**Figure 11A**). TIMER database analysis results suggested that the copy number of ESPL1 was significantly correlated with the TILs in LUAD (**Figure 11B**). We used the ssGSEA method to analyze the correlation between ESPL1 expression and immune infiltration. Results indicated that ESPL1 expression was positively correlated with the infiltration levels of Th2 cells, and negatively associated with infiltration levels of mast cells, iDC, DC, and CD8 T cells in LUAD (**Figures 11C–E**).

**Figure 12:**
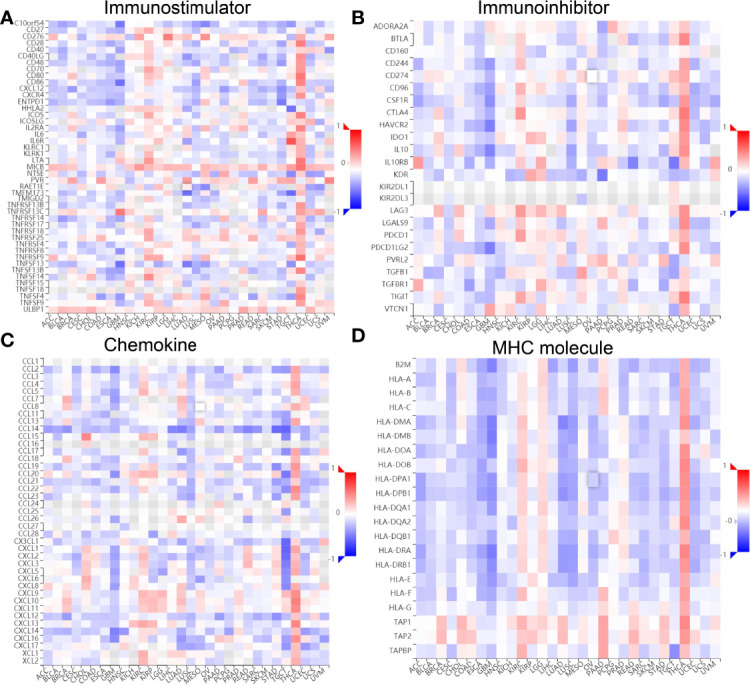
Correlation between ESPL1 expression and immune modulator. **(A–D)** Examine the relationship between ESPL1 expression, immunostimulators, immunoinhibitors, chemokine, and MHC molecule by using the TISIDB database.

Finally, we examined the relationship between ESPL1 expression, immunostimulators, immunoinhibitors, chemokines, and MHC molecules by using the TISIDB database. Spearman’s correlation analysis results suggested that ESPL1 expression was negatively correlated with immunostimulators (CD40LG, HHLA2, TMEM173, and tnfsf13), immunoinhibitors (KD2 and TGFB1), chemokines (CCL14, CCL17, CCL19, CXCL1, CXCL16, and CXCL17), and MHC molecules (HLA-DMA, HLA-DMB, HLA-DOA, and HLA-DOB) in LUAD (**Figures 12A–D**).

## Discussion

Tumor microenvironment (TME) plays an important role in the dynamic regulation of tumor progression, and strategies to therapeutically target the TME have emerged as a promising approach for cancer treatment (22). Immunotherapy, which has been approved or is being evaluated in clinical trials, has a wide application prospect (23). A comprehensive analysis of tumor-infiltrating immune cells will help to clarify the mechanism of tumor immune escape and provide opportunities for the development of new therapeutic strategies. ESPL1 is an etiological factor that regulates many biological processes, such as hematopoietic differentiation and cell proliferation (7). Nevertheless, there is still no research examining whether ESPL1 is correlated with LUAD progression or whether it can be a prognostic and diagnostic biomarker for LUAD.

In this study, we first analyzed ESPL1 expression in diverse cancers. We found that ESPL1 expression was significantly higher in BLCA, BRCA, CESC, CHOL, COAD, ESCA, GBM, LIHC, LUAD, LUSC, PRAD, READ, and UCEC. Higher expression of ESPL1 was correlated with adverse OS in ACC, KIRP, LGG, LIHC, MEAO, PAAD, SARC, and SKCM.

It has been confirmed that ESPL1 was highly expressed as a prognostic biomarker in glioma and LIHC (7, 8). In our study, we found that ESPL1 was upregulated in LUAD and LUSC, and clinical feature analysis suggested that higher expression of ESPL1 was significantly correlated with higher clinical stage, TNM stage, gender, age, residual tumor, smoker, OS event, and DSS event in patients with LUAD. The Kaplan–Meier curve shows that patients in the higher ESPL1 class had a lower probability of OS, DSS, and PFS compared to the low ESPL1 group. Then, ROC analysis suggested that the ESPL1 may be used to differentiate LUAD patients from normal control. Univariate regression analysis and a multivariate model have shown significant prognostic significance of ESPL1 expression, pathological stage, and TNM stage for OS and DSS in LUAD.

Studies have shown that higher ESPL1 expression was associated with poor prognosis and advanced stage in luminal B breast cancers, and it as a candidate oncogene for BRCA (24). In our study, we confirmed that ESPL1 was upregulated in lung cancer and cell lines, which is consistent with the online database we discovered. We showed that downregulation of ESPL1 significantly decreased the migratory and invasion capabilities of LUAD cells. Collectively, these results confirmed that ESPL1 was highly expressed in LUAD cells and significantly affected their migration and invasion. In addition, it was reported that c-MYB is a crucial transcriptional regulator that modulates the expression of ESPL1 in chronic myeloid leukemia (25). In this study, we show that DNA copy amplification and DNA hypo-methylation were two vital mechanisms of ESPL1 upregulation and were associated with poor prognosis.

Previously, several studies have reported that ESPL1 mainly enriched in the cell cycle pathway in endometrial cancer (9). In our study, we found that ESPL1 was mainly involved in apoptosis, p53 signaling pathway, IL6-JAK-STAT3 signaling pathway, IL3-STAT5 signaling pathway, MYC targets, hypoxia, glycolysis, TNFα signaling pathway, G2/M checkpoint, fatty acid metabolism, PI3K-AKT signaling pathway, EMT, and DNA repair.

Increasing lines of evidence have shown that TME plays an important role in cancer metastasis, immune escape, and immunotherapy resistance (26, 27). In our findings, we revealed that ESPL1 expression was significantly upregulated in the C2 subtype of LUAD. TIMER database analysis results suggested that the copy number of ESPL1 was significantly correlated with the TILs in LUAD. Upregulated ESPL1 expression was positively correlated with the infiltration levels of Th2 cells, and negatively associated with infiltration levels of mast cells, iDC, DC, and CD8 T cells in LUAD. Finally, we show that ESPL1 expression was negatively correlated with immunostimulators (CD40LG, HHLA2, TMEM173, and tnfsf13), immunoinhibitors (KD2 and TGFB1), chemokines (CCL14, CCL17, CCL19, CXCL1, CXCL16, and CXCL17), and MHC molecules (HLA-DMA, HLA-DMB, HLA-DOA, and HLA-DOB) in LUAD.

## Conclusion

Together, these findings suggest that ESPL1 is a valuable biomarker for prognosis and is significantly correlated with immune infiltration in LUAD.

## Data Availability Statement

The original contributions presented in the study are included in the article/supplementary material. Further inquiries can be directed to the corresponding authors.

## Author Contributions

ZN, TP, ZH, CheW, and CP designed this work and performed related assay. PL, XM, YY, YZ, analyzed the data. JD, ChuW and XJ supervised and wrote the manuscript. All authors have read and approved the final version of the manuscript.

## Funding

This study was supported in part by grants from the National Natural Science Foundation of China (82160461 to CW), the Department of Science and Technology of Yunnan Province-Kunming Medical University [2019FE001(-063) to CW], and Yunnan Health Training Project of High Level Talents (D2018058 to CW).

## Conflict of Interest

The authors declare that the research was conducted in the absence of any commercial or financial relationships that could be construed as a potential conflict of interest.

## Publisher’s Note

All claims expressed in this article are solely those of the authors and do not necessarily represent those of their affiliated organizations, or those of the publisher, the editors and the reviewers. Any product that may be evaluated in this article, or claim that may be made by its manufacturer, is not guaranteed or endorsed by the publisher.

## Abbreviations

ACC, Adrenocortical carcinoma; BLCA, Bladder Urothelial Carcinoma; BRCA, Breast invasive carcinoma; CESC, Cervical squamous cell carcinoma and endocervical adenocarcinoma; CHOL, Cholangiocarcinoma; COAD, Colon adenocarcinoma; DLBC, Lymphoid Neoplasm Diffuse Large B-cell Lymphoma; ESCA, Esophageal carcinoma; GBM, Glioblastoma multiforme; HNSC, Head and Neck squamous cell carcinoma; KICH, Kidney Chromophobe; KIRC, Kidney renal clear cell carcinoma; KIRP, Kidney renal papillary cell carcinoma; LAML, Acute Myeloid Leukemia; THCA, Thyroid carcinoma; UCEC, Uterine Corpus Endometrial Carcinoma; UCS, Uterine Carcinosarcoma; LGG, Brain Lower Grade Glioma; LIHC, Liver hepatocellular carcinoma; LUAD, Lung adenocarcinoma; LUSC, Lung squamous cell carcinoma; MESO, Mesothelioma; OV, Ovarian serous cystadenocarcinoma; PAAD, Pancreatic adenocarcinoma; PCPG, Pheochromocytoma and Paraganglioma; PRAD, Prostate adenocarcinoma; READ, Rectum adenocarcinoma; SARC, Sarcoma; SKCM, Skin Cutaneous Melanoma; STAD, Stomach adenocarcinoma; TGCT, Testicular Germ Cell Tumors; THYM, Thymoma; UVM, Uveal Melanoma.

## References

[1] Siegel RL Miller KD Fuchs HE Jemal A. Cancer Statistics, 2022. CA Cancer J Clin (2022) 72(1):7–33. doi: 10.3322/caac.21708 35020204

[2] Siegel RL Miller KD Jemal A. Cancer Statistics, 2020. CA: A Cancer J Clin (2020) 70(1):7–30. doi: 10.3322/caac.21590 31912902

[3] Molina JR Yang P Cassivi SD Schild SE Adjei AA. Non-Small Cell Lung Cancer: Epidemiology, Risk Factors, Treatment, and Survivorship. Mayo Clin Proc (2008) 83(5):584–94. doi: 10.1016/S0025-6196(11)60735-0 PMC2718421 18452692

[4] Herbst RS Morgensztern D Boshoff C. The Biology and Management of non-Small Cell Lung Cancer. Nature (2018) 553(7689):446–54. doi: 10.1038/nature25183 29364287

[5] Lee SS Cheah YK. The Interplay Between MicroRNAs and Cellular Components of Tumour Microenvironment (TME) on Non-Small-Cell Lung Cancer (NSCLC) Progression. J Immunol Res (2019) 2019:3046379. doi: 10.1155/2019/3046379 30944831 PMC6421779

[6] Chestukhin A Pfeffer C Milligan S DeCaprio JA Pellman D. Processing, Localization, and Requirement of Human Separase for Normal Anaphase Progression. Proc Natl Acad Sci U S A (2003) 100(8):4574–9. doi: 10.1073/pnas.0730733100 PMC153597 12672959

[7] Liu Z Lian X Zhang X Zhu Y Zhang W Wang J. ESPL1 Is a Novel Prognostic Biomarker Associated With the Malignant Features of Glioma. Front Genet (2021) 12:666106. doi: 10.3389/fgene.2021.666106 34512713 PMC8428966

[8] Wang R Zang W Hu B Deng D Ling X Zhou H. Serum ESPL1 Can Be Used as a Biomarker for Patients With Hepatitis B Virus-Related Liver Cancer: A Chinese Case-Control Study. Technol Cancer Res Treat (2020) 19:1533033820980785. doi: 10.1177/1533033820980785 33308056 PMC7739072

[9] Yang Q Yu B Sun J. TTK, CDC25A, and ESPL1 as Prognostic Biomarkers for Endometrial Cancer. BioMed Res Int (2020) 2020:4625123. doi: 10.1155/2020/4625123 33282948 PMC7685798

[10] Cerami E Gao J Dogrusoz U Gross BE Sumer SO Aksoy BA. The Cbio Cancer Genomics Portal: An Open Platform for Exploring Multidimensional Cancer Genomics Data. Cancer Discovery (2012) 2(5):401–4. doi: 10.1158/2159-8290.CD-12-0095 PMC3956037 22588877

[11] Colwill K Gräslund S. A Roadmap to Generate Renewable Protein Binders to the Human Proteome. Nat Methods (2011) 8(7):551–8. doi: 10.1038/nmeth.1607 21572409

[12] Mizuno H Kitada K Nakai K Sarai A. PrognoScan: A New Database for Meta-Analysis of the Prognostic Value of Genes. BMC Med Genomics (2009) 2:18. doi: 10.1186/1755-8794-2-18 19393097 PMC2689870

[13] Crosara KTB Moffa EB Xiao Y Siqueira WL. Merging *in-Silico* and *In Vitro* Salivary Protein Complex Partners Using the STRING Database: A Tutorial. J Proteomics (2018) 171:87–94. doi: 10.1016/j.jprot.2017.08.002 28782718

[14] Warde-Farley D Donaldson SL Comes O Zuberi K Badrawi R Chao P. The GeneMANIA Prediction Server: Biological Network Integration for Gene Prioritization and Predicting Gene Function. Nucleic Acids Res (2010) 38(Web Server issue):W214–20. doi: 10.1093/nar/gkq537 PMC2896186 20576703

[15] Yuan H Yan M Zhang G Liu W Deng C Liao G. CancerSEA: A Cancer Single-Cell State Atlas. Nucleic Acids Res (2019) 47(D1):D900–d908. doi: 10.1093/nar/gky939 30329142 PMC6324047

[16] Liu CJ Hu FF Xia MX Han L Zhang Q Guo AY. GSCALite: A Web Server for Gene Set Cancer Analysis. Bioinformatics (2018) 34(21):3771–2. doi: 10.1093/bioinformatics/bty411 29790900

[17] Chandrashekar DS Bashel B Balasubramanya SAH Creighton CJ Ponce-Rodriguez I Chakravarthi B. UALCAN: A Portal for Facilitating Tumor Subgroup Gene Expression and Survival Analyses. Neoplasia (2017) 19(8):649–58. doi: 10.1016/j.neo.2017.05.002 PMC5516091 28732212

[18] Li T Fan J Wang B Traugh N Chen Q Liu JS. TIMER: A Web Server for Comprehensive Analysis of Tumor-Infiltrating Immune Cells. Cancer Res (2017) 77(21):e108–10. doi: 10.1158/0008-5472.CAN-17-0307 PMC6042652 29092952

[19] Bindea G Mlecnik B Tosolini M Kirilovsky A Waldner M Obenauf AC. Spatiotemporal Dynamics of Intratumoral Immune Cells Reveal the Immune Landscape in Human Cancer. Immunity (2013) 39(4):782–95. doi: 10.1016/j.immuni.2013.10.003 24138885

[20] Yuan Y Jiang X Tang L Wang J Zhang D Cho WC. FOXM1/lncRNA TYMSOS/miR-214-3p-Mediated High Expression of NCAPG Correlates With Poor Prognosis and Cell Proliferation in Non-Small Cell Lung Carcinoma. Front Mol Biosci (2021) 8:785767. doi: 10.3389/fmolb.2021.785767 35211508 PMC8862726

[21] Kulis M Esteller M. DNA Methylation and Cancer. Adv Genet (2010) 70:27–56. doi: 10.1016/B978-0-12-380866-0.60002-2 20920744

[22] Uprety D Mandrekar SJ Wigle D Roden AC Adjei AA. Neoadjuvant Immunotherapy for NSCLC: Current Concepts and Future Approaches. J Thorac Oncol (2020) 15(8):1281–97. doi: 10.1016/j.jtho.2020.05.020 32522713

[23] Ruiz-Cordero R Devine WP. Targeted Therapy and Checkpoint Immunotherapy in Lung Cancer. Surg Pathol Clin (2020) 13(1):17–33. doi: 10.1016/j.path.2019.11.002 32005431

[24] Finetti P Guille A Adelaide J Birnbaum D Chaffanet M Bertucci F. ESPL1 is a Candidate Oncogene of Luminal B Breast Cancers. Breast Cancer Res Treat (2014) 147(1):51–9. doi: 10.1007/s10549-014-3070-z 25086634

[25] Prinzhorn W Stehle M Kleiner H Ruppenthal S Müller MC Hofmann WK. C-MYB is a Transcriptional Regulator of ESPL1/Separase in BCR-ABL-Positive Chronic Myeloid Leukemia. biomark Res (2016) 4:5. doi: 10.1186/s40364-016-0059-2 26937281 PMC4774018

[26] Darragh LB Oweida AJ Karam SD. Overcoming Resistance to Combination Radiation-Immunotherapy: A Focus on Contributing Pathways Within the Tumor Microenvironment. Front Immunol (2018) 9:3154. doi: 10.3389/fimmu.2018.03154 30766539 PMC6366147

[27] Chen F Zhuang X Lin L Yu P Wang Y Shi Y. New Horizons in Tumor Microenvironment Biology: Challenges and Opportunities. BMC Med (2015) 13:45. doi: 10.1186/s12916-015-0278-7 25857315 PMC4350882

